# Knockdown of the *Rhipicephalus microplus* Cytochrome *c* Oxidase Subunit III Gene Is Associated with a Failure of *Anaplasma marginale* Transmission

**DOI:** 10.1371/journal.pone.0098614

**Published:** 2014-05-30

**Authors:** Thais D. Bifano, Massaro W. Ueti, Eliane Esteves, Kathryn E. Reif, Glória R. C. Braz, Glen A. Scoles, Reginaldo G. Bastos, Stephen N. White, Sirlei Daffre

**Affiliations:** 1 Department of Parasitology, Instituto de Ciências Biomédicas, Universidade de São Paulo, São Paulo, SP, Brazil; 2 Department of Veterinary Microbiology and Pathology, Washington State University, Pullman, Washington, United States of America; 3 Animal Diseases Research Unit, USDA-ARS, Pullman, Washington United States of America; 4 Department of Biochemistry, Instituto de Química, Universidade Federal do Rio de Janeiro, Rio de Janeiro, RJ, Brazil; 5 Instituto Nacional de Ciência e Tecnologia em Entomologia Molecular, Rio de Janeiro, RJ, Brazil; Kansas State University, United States of America

## Abstract

*Rhipicephalus microplus* is an obligate hematophagous ectoparasite of cattle and an important biological vector of *Anaplasma marginale* in tropical and subtropical regions. The primary determinants for *A. marginale* transmission are infection of the tick gut, followed by infection of salivary glands. Transmission of *A. marginale* to cattle occurs via infected saliva delivered during tick feeding. Interference in colonization of either the tick gut or salivary glands can affect transmission of *A. marginale* to naïve animals. In this study, we used the tick embryonic cell line BME26 to identify genes that are modulated in response to *A. marginale* infection. Suppression-subtractive hybridization libraries (SSH) were constructed, and five up-regulated genes {glutathione S-transferase (GST), cytochrome *c* oxidase sub III (COXIII), dynein (DYN), synaptobrevin (SYN) and phosphatidylinositol-3,4,5-triphosphate 3-phosphatase (PHOS)} were selected as targets for functional *in vivo* genomic analysis. RNA interference (RNAi) was used to determine the effect of tick gene knockdown on *A. marginale* acquisition and transmission. Although RNAi consistently knocked down all individually examined tick genes in infected tick guts and salivary glands, only the group of ticks injected with dsCOXIII failed to transmit *A. marginale* to naïve calves. To our knowledge, this is the first report demonstrating that RNAi of a tick gene is associated with a failure of *A. marginale* transmission.

## Introduction

Ticks and tick-borne pathogens, including *Anaplasma marginale*, cause significant economic losses for the livestock industry worldwide. These economic losses are associated with the following: 1) reduction of milk and meat production; 2) temporary infertility; 3) treatment costs; 4) mortality and 5) secondary bacterial infection in the open wounds caused by tick feeding [1]. *Anaplasma marginale* is an obligate gram-negative bacterium transmitted by ticks, including *Rhipicephalus* species. In Latin America, it is estimated that bovine anaplasmosis and babesiosis cause annual economic losses exceeding US$ 800 million [2]. In endemic regions, anaplasmosis control strategies include the use of a live-attenuated vaccine, a killed vaccine, antibiotic prophylaxis and/or tick control measures [3], [4]. Vaccines are the most effective method for controlling disease and induce protective immunity that prevents acute bacteremia. However, vaccines do not prevent *A. marginale* infection, and infected animals can serve as reservoirs for tick transmission [1], [4].

Ticks are an efficient biological vector of *A. marginale* and acquire the bacteria from acutely or persistently infected animals [5]. There is no transovarial transmission of *A. marginale* from female ticks to tick offspring [6], [7], and transstadial and intrastadial transmission by male ticks are considered the most important means of *A. marginale* transmission [8], [9]. In the tick, *A. marginale* first infects gut epithelial cells. After colonization of the tick gut, the bacteria migrate through the hemocoel to infect tick salivary glands [10]. Transmission occurs via saliva when infected ticks feed on an uninfected host [11].

Cellular and molecular interactions between *A. marginale* and ticks are poorly understood. Tick cell lines, including ISE6, IDE8 (derived from *Ixodes scapularis*) and BME26 (derived from *R. microplus*), have been used in transcriptional and protein expression studies to examine differential tick responses to *A. marginale* infection [12]–[16]. Those studies demonstrated that *A. marginale* infection alters normal tick gene transcription and protein expression. In the current study, we identified differentially regulated tick genes in response to *A. marginale* infection in a BME26 cell line by suppression-subtractive hybridization. A subset of differentially regulated tick genes was selected based on functional annotation and targeted for *in vivo* gene knockdown studies using RNAi. We examined the impact of *R. microplus* gene knockdown on *A. marginale* acquisition and transmission.

## Materials and Methods

### Ethics Statement

All experiments involving animals were approved by the University of Idaho, Institutional Animal Care and Use and Biosafety Committees (Protocol Numbers, IACUC: 2013-66, Biosafety: B-010-13) in accordance with institutional guidelines based on the U.S. National Institutes of Health (NIH) Guide for the Care and Use of Laboratory Animals.

### Cattle infection by *Anaplasma marginale* and male tick rearing

Eleven spleen-intact, age-matched (5-month old) Holstein calves were used in this study: two for rearing ticks (C38080 and C40440), two for acquisition feeding experiments (C37837 and C39306) and seven for transmission feeding experiments (C38098, C38099, C38100, C38101, C38118, C40444 and C40456). These calves were confirmed to be free of *A. marginale* by MSP5-CI-ELISA [17] and *msp5*-nested PCR [18]. The calves used in the acquisition feeding experiments were inoculated with ∼10^8^ *A. marginale*-infected erythrocytes (St. Maries strain) as described previously [11].

To rear male ticks, approximately 40,000 larvae from 2.0 grams of *R. microplus* eggs were placed under a cloth patch on a naïve calf. On day 14, engorged nymphs were manually removed from the calf with forceps and held in an incubator at 26°C and 92% relative humidity until molting into adults.

### Infection of BME26 with *Anaplasma marginale*

BME26, an embryonic cell line derived from the cattle fever tick, *Rhipicephalus microplus*, was cultured in L-15B300 medium as previously described [19]. Approximately 3.5×10^6^ cells were sub-cultured into eight new flasks and incubated for 24 h, after which time 5 ml of the culture medium in each flask was replaced with the same volume of *Anaplasma* culture medium [20]. After seven days, four flasks were sub-inoculated with 25 day-old *A. marginale* (Brazilian strain UFMG2) culture (31% infected cells). After 72 h, cells were collected from culture flasks and transferred to sterile polypropylene tubes. The tubes containing the *A. marginale*-infected cell suspensions were centrifuged at 800xg for 15 min, and both RNA and genomic DNA were isolated using TRIZOL Reagent (Invitrogen, San Diego, CA, USA) following the manufacturer's instructions.

### Identification of differentially regulated tick genes during *A. marginale* infection

Suppression-subtractive hybridization (SSH) was performed using the Clontech PCR-Select cDNA Subtraction Kit [21], and cDNA was prepared using the SMARTer Pico PCR cDNA Synthesis Kit, according to the manufacturer's instructions (Clontech, Palo Alto, CA, USA). To identify tick genes that are up-or down-regulated as a consequence of *A. marginale* infection, forward and reverse SSH libraries were constructed as follows: pools of 2 µg total RNA were prepared from uninfected and *A. marginale*-infected BME26 cells. After *Rsa*l digestion, cDNA from both groups were ligated to adaptors. The forward SSH library was made by hybridizing adapter ligated cDNA from BME26 cells infected with *A. marginale* as the tester in the presence of an excess of cDNA from uninfected cells as the driver. The reverse SSH library was made in the same manner, but in this case, the adapter ligated cDNA from uninfected BME26 cells was used as the tester, and infected cells cDNA as the driver. The forward and reverse libraries were used to identify up- or down-regulated BME26 transcripts, respectively, in response to *A. marginale* infection. Differentially expressed cDNAs were PCR amplified with Advantage PCR Polymerase Mix (Clontech), cloned using the pGEM-T Easy Vector System (Promega, Madison, WI, USA), and transformed into XL1-Blue *E. coli* cells plated on LB with ampicillin, X-gal and IPTG. Individual colonies were randomly selected from each library and inoculated into LB medium supplemented with ampicillin and incubated overnight. Plasmids were purified using the Wizard SV 96 Plasmid DNA Purification System (Promega), and plasmid inserts were PCR amplified and sequenced on an ABI PRISM 3700 DNA sequencer (Applied Biosystems, Foster City, CA, USA) using the BigDye Terminator cycle sequencing protocol (Applied Biosystems).

### Functional annotation of assembled sequences

High quality sequences were obtained from both the forward and reverse SSH libraries and assembled together in one run into unique contigs by the CAP3 assembler (Contig Assembly Program, Version 3) as previously described [22]. The library origin of the reads assembled in each up- or down-regulated contig was tracked, and except in the case of glutathione S-transferase, the contigs assembled from reads from only one library. Both manual and automatic annotations were performed to search for similarity among the sequences obtained in both SSH libraries and other libraries deposited in public databases (GenBank and Swiss-Prot), as well as to all sequences available from *R. microplus*. For these purpose two data banks of DNA sequences were made: one created from public data of *R. microplus* (GenBank) ESTs (RHIPI2011 database), and another constructed after the analysis of an extensive transcriptome (seven organs/tissues of *R. microplus*, harvested in various stages of development) (Solexa-ASB database). In this last case, the cDNA generated was sequenced by Illumina technology, and the complete analysis of the results obtained will be the subject of another publication. To search for conserved functions, the sequences were analyzed by RPS Blast against several conserved domain databases, including GO (Gene Ontology) [23], SignalP (Signal Peptide) [24], KOG (EuKaryotic Orthologous Groups) [25], CDD (Conserved Domain Databases) [26], PFAM (Protein family database) [27], SMART (Simple Modular Architecture Research Tool) [28] and MIT-PLA (Mitochondrial and Plasmid Sequences database), available from NCBI. The final assembly output was piped into a tab-delimited file that was imported into an Excel spreadsheet, which includes functional classification of each assembled up and down contigs, as described previously [29].

### Quantification of tick gene expression and *A. marginale* in *R. microplus* guts and salivary glands

Total RNA and genomic DNA was extracted from pools of 5 adult male tick guts and salivary glands using AllPrep DNA/RNA Micro (QIAGEN, Valencia, CA, USA) according to the manufacturer's instructions. Total RNA samples were treated with DNase I (Invitrogen), and cDNA was synthesized with AllPrep DNA/RNA Micro (Bio-Rad, Hercules, CA, USA).

To quantify the tick gene expression, real time quantitative PCR (qPCR) was performed on a CFX96 Real-Time PCR Detection System (Bio-Rad) and Express SYBR Green ER Supermix (Invitrogen), using cDNA as template and specific primers (Table S1). The qPCR cycling conditions consisted of an initial denaturation of 95°C for 30 sec followed by 40 cycles at 95°C denaturation for 5 sec, and annealing/extension at 60°C for 10 sec. Reactions were performed in triplicate in 20 µl volumes using 5 µM of each primer and 2 µl of a 1/20 dilution of cDNA as template. The CFX Manager Software (Bio-Rad) was used to analyze relative gene expression data. The threshold cycle (Ct) was determined, and the relative quantification method Delta Delta Ct [30] was used to calculate the relative expression of the target gene after normalization to the reference gene *R. microplus* tubulin gene, as previously described [31]. The 2-ΔΔCt equation was applied to calculate the relative expression of selected genes in experimental versus control samples, where ΔCt = Ct gene - Ct tubulin and ΔΔCt = ΔCt infected - mean ΔCt control (uninfected) samples or ΔΔCt = ΔCt dsRNA samples - mean ΔCt control (0.1 mM buffered EDTA) samples [24].

The total number of *Anaplasma* in tick guts and salivary glands were quantified by qPCR using gDNA as template, specific primers (Table S1) and a TaqMan probe targeting *msp5* [18]. Briefly, the reactions were performed on a CFX96 Real-Time PCR Detection System (Bio-Rad) using the following thermocycler program: 10 minutes at 95°C followed by 55 cycles of 15 seconds at 95°C and 45 seconds at 55°C. Three technical replicates were analyzed for each sample. The CFX Manager Software (Bio-Rad) was used to analyze bacteria quantification data.

### Preparation of double-stranded RNA

Glutathione S-transferase (GST), cytochrome *c* oxidase subunit III (COXIII), dynein (DYN), synaptobrevin (SYN) and phosphatidylinositol-3,4,5-triphosphate 3-phosphatase (PHOS) were selected for knockdown studies using RNA interference technique. Primer sets for each gene were designed (Table S1) using Primer3 software [32], [33]. Tick guts and salivary glands from adult males were used as a template for generating target gene amplicons. Amplicons were cloned into the TOPO TA Cloning Kit Dual Promoter (with pCRII-TOPO) (Invitrogen). The MEGAscript Transcription Kit was used for dsRNA synthesis following the manufacturer's protocol (Ambion, Austin, TX, USA). The dsRNA molecules were purified, quantified by spectrophotometry, analyzed by gel electrophoresis, and stored at −20°C.

### Transmission trials of *A. marginale*

Five cohorts of 200 freshly molted male ticks for each experimental group were injected with dsGST, dsCOXIII, dsDYN+SYN, dsPHOS and buffered 0.1 mM EDTA in trial 1. Ticks were placed ventral side up on double-sided tape for injections. They were injected with 2×10^11^ molecules of dsRNA suspended in buffered 0.1 mM EDTA through the coxal membrane at the base of the 4^th^ leg on the right ventral side using a Hamilton syringe with a 36 gauge needle and a microprocessor-controlled UMP3 injection pump apparatus (World Precision Instruments, San Antonio, TX, USA) [34], [35]. The control group was injected with 0.1 mM buffered EDTA in a similar manner. After injection, male ticks were immediately placed in individual group patches on an infected calf (C37837) and allowed to acquisition feed for 8 days during peak bacteremia. Ticks were removed and held at 26°C for 24 h to allow for the clearance of the blood meal from their mouthparts. The survival rate was evaluated by the number of attached ticks x 100/200 ticks. The surviving ticks were transferred by group to naïve recipient calves for transmission feeding as follows: C38098 received ticks injected with EDTA buffer; C38099 received dsGST injected ticks; C38100 received dsCOXIII injected ticks; C38101 received dsDYN+SYN injected ticks; and C38118 received dsPHOS injected ticks. After 7 days of transmission feeding, ticks were removed and the survival rate was evaluated by the number of attached ticks x 100/placed ticks on each experimental group. The RNA and genomic DNA from both gut and salivary glands were collected for gene expression and bacteria quantification by RT-qPCR and qPCR, respectively. After removal of all ticks, recipient calves were monitored weekly for up to 12 weeks post-tick transmission feeding. Infection was assessed by Giemsa stained blood smears [11], nested PCR [18] and serology [17].

In trial 2, two cohorts of 150 freshly molted male ticks for each experimental group were injected with dsCOXIII and EDTA buffer and placed on an *Anaplasma-*infected calf (C39306) during peak bacteremia. Calves C40444 and C40456 were used for tick transmission-feeding experiments. All analyses were made following the same methodology described for trial 1.

### Statistical analysis

*A. marginale* infection and tick gene expression data were analyzed by Student's t-test using SAS 9.2 (SAS Institute, Cary, NC, USA). If an initial test for equality of variances was not significant, then a t-test was performed. However, if this initial test was significant (P≤0.05), then a Satterthwaite t-test was performed [28]. Tick survival data were analyzed by chi-square tests unless any contingency table cell contained fewer than 5, in which case Fisher's exact test was used instead. The results are expressed as the mean ± S.D. P value≤0.05 was considered significant.

## Results

### Identification of differentially expressed tick genes during *A. marginale* infection

**Figure 1:**
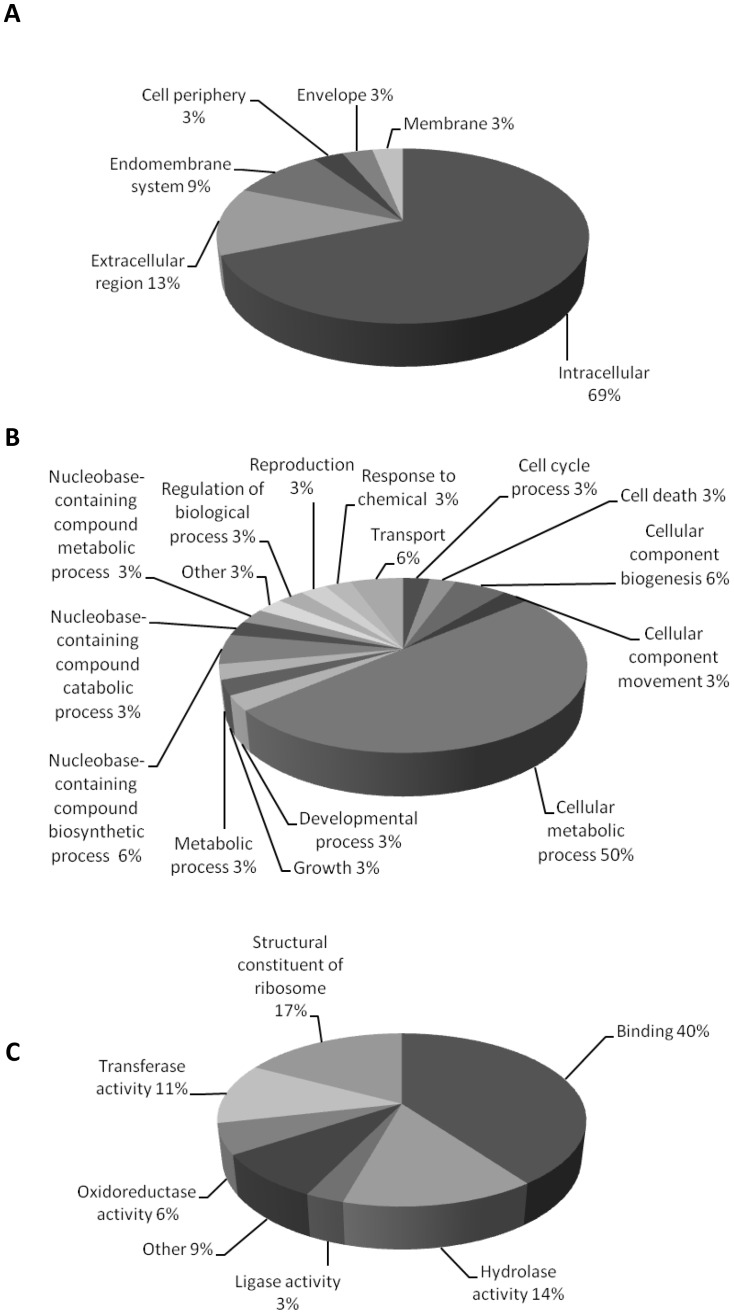
The Gene Ontology analysis. Sequences of up-regulated genes identified by SSH were categorized by (A) cellular component, (B) molecular function and (C) biological process.

Forward and reverse SSH libraries were constructed to identify up- or down-regulated tick genes in response to *A. marginale* infection using a BME26 cell culture, and 1,536 randomly selected clones were sequenced. After eliminating clones with poor quality sequences, 719 expressed sequence tags (EST) were obtained and used for bioinformatics analysis (Table 1). Clustering and assembly of ESTs from up-regulated genes resulted in 25 contigs and 106 singletons. Down-regulated genes yielded 211 unique sequences with 13 contigs sequences and 85 singletons. Automated and manual annotation were used to search databases (GenBank, Swiss-Prot, RHIPI2011 and Solexa-ASB) for similarity and putative functions (GO, SignalP, KOG, CDD, PFAM, SMART and MIT-PLA), and a hyperlinked excel spreadsheet was prepared with the various information obtained for each contig (Table S2). The sequences are identified as Rm-contig_number in the first column, which is hyperlinked to its FASTA nucleotide sequence. Gene ontology assignments were used to obtain more information about up-regulated genes from the forward SSH library, and sequences were broadly split into categories of ‘cellular component’, ‘molecular function’ or ‘biological process’. A total of 33 sequences were categorized as cellular components (Figure 1A), 35 sequences were assigned to molecular functions (Figure 1B) and 36 sequences were assigned to biological processes (Figure 1C). In the cellular component category, the majority of transcripts (69%) were classified as intracellular, and 13% were classified as extracellular. For biological process, the majority of identified transcripts were related to a cellular metabolic process (50%). In the molecular function category, most transcripts were related to binding (40%), catalytic activity (34%), transferase activity (11%), oxidoreductase activity (6%), ligase activity (3%) or hydrolase activity (14%).

**Table 1:** Summary of up- and down-regulated genes from *A. marginale*-infected BME26 cells.

Library	Up-regulated	Down-regulated
Total sequences	508	211
Non-redundant sequences:		
Number of singletons	106	85
Number of contigs	25	13
Number of contigs containing:		
2–4 sequences	12	8
5–15 sequences ESTs	8	4
>15 sequences	5	1

### Selection of tick genes for the genomic functional study

We elected to study five genes with increased transcription during *A. marginale* infection in BME26 cells in an *in vivo* model. The selection of these tick genes was based on biological functions that could be related to *Anaplasma* colonization in tick cells. These genes include one isoform of glutathione S-transferase (GST) (GenBank accession number KF784792), cytochrome *c* oxidase sub III (COXIII) (GenBank accession number KF784795), dynein (DYN) (GenBank accession number KF784791), synaptobrevin (SYN) (GenBank accession number KF784794) and phosphatidylinositol-3,4,5-triphosphate 3-phosphatase (PHOS) (GenBank accession number KF784793). GST is important in the detoxification of both endogenous and xenobiotic compounds and protection against oxidative stress [36]–[38]; COXIII is related to mitochondrial metabolism, including ATP production and reactive oxygen species (ROS); DYN is a motor protein involved in the conversion of chemical energy present in molecules of ATP into mechanical energy along microtubules and is involved in intracellular trafficking pathways [39]; SYN is a v-SNARE protein that participates in the exocytosis of proteins [40]–[42]; and phosphatidylinositol-3,4,5-trisphosphate 3-phosphatase plays an important role in the reorganization of the cytoskeleton, NADPH production and ROS production [43].

**Figure 2:**
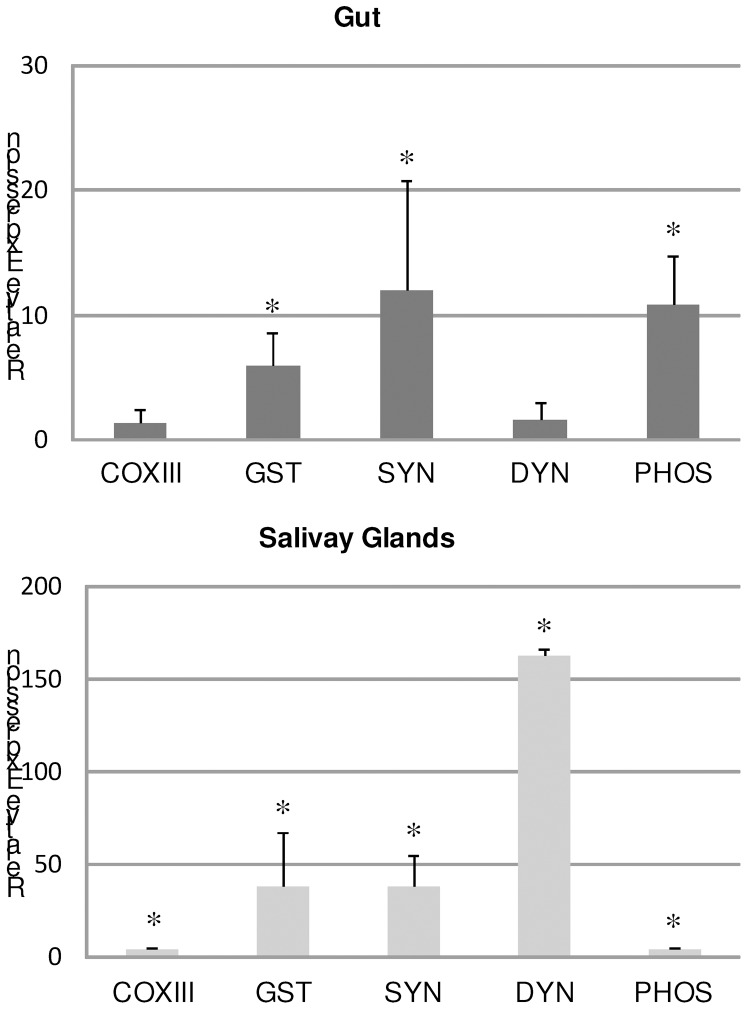
Relative gene expression in gut and salivary glands of ticks infected with *A. marginale*. The expression of the cytochrome *c* oxidase sub III (COXIII), glutathione S-transferase (GST), synaptobrevin (SYN), dynein (DYN) and phosphatidylinositol-3,4,5-triphosphate 3-phosphatase (PHOS) genes in guts and salivary glands from *R. microplus* males fed for 8 days on either one uninfected calf (C38080) or one *A. marginale*-infected calf (C37837) was assessed by RT-qPCR. Threshold values were normalized according to the Ct of the reference gene (tubulin). The relative expression level of each gene in infected ticks in relation to uninfected ticks (control) was calculated using the Delta Delta Ct method. The data represent the mean ± S.D. of four pools of 5 guts and salivary glands. An asterisk (*) represent data with differences statistically significant with respect to control (*P*≤0.05).

In order to confirm the up-regulation of the selected genes from the forward SSH library, the relative gene expression in uninfected and *A. marginale*-infected ticks (harboring 10^3.35±0.52^ bacteria per gut and 10^1.93±0.46^ bacteria per salivary gland pair) was determined (Figure 2). We observed that GST, COXIII, DYN, SYN and PHOS genes were significantly up-regulated in tick gut and salivary glands during *A. marginale* infection, with the exception of DYN and COXIII in the gut.

### Effect of tick gene knockdown during transmission of *A. marginale*

#### Trial 1

In order to evaluate the outcome of tick gene knockdown on *Anaplasma* acquisition and transmission to calves, RNAi was performed. Groups of freshly molted male ticks were injected with dsGST, dsCOXIII, dsDYN+SYN, dsPHOS and buffered 0.1 mM EDTA. DYN and SYN were injected together because both are related to vesicular trafficking. After injection, male ticks were immediately placed in individual group patches on an infected calf (C37837) and allowed to acquisition feed for 8 days during peak bacteremia. Calf peak bacteremia ranged from 4.2% to 13.6%, and the packed cell volume (PCV) varied from 13% to 38%. The tick survival rate evaluated for the five tick groups at the end of acquisition feeding varied from 30% to 60.5% (Table 2). There was a significant difference in tick survival rates between the dsRNA-injected groups and the control group, in which fewer ticks survived (30%).

**Table 2:** Survival rate of dsRNA injected ticks.

	Target gene	Tick acquisition feeding	Tick acquisition feeding
	GST	*51% (102^a^/200^b^)	94% (77^a^/82^b^)
	COXIII	*59% (118^a^/200^b^)	*56% (55^a^/98^b^)
**Trial 1**	DYN + SYN	*60.5% (121^a^/200^b^)	*74% (75^a^/101^b^)
	PHOS	*46.5% (93^a^/200^b^)	82% (60^a^/73^b^)
	Control	30% (60^a^/200^b^)	93% (37^a^/40^b^)
**Trial 2**	COXIII	41% (61^a^/150^b^)	88% (36^a^/41^b^)
	Control	39% (59^a^/150^b^)	79% (31^a^/39^b^)

Survival rate was calculated by the number of attached ticks ^(a)^ x 100/placed ticks ^(b)^ in calves. Asterisk (*) indicates significant difference (*P*≤0.05) between dsRNA injected and control ticks.

The surviving male ticks injected with dsRNA were allowed to transmission feed on naïve calves for seven days. At the end of transmission feeding, the tick survival rates varied from 56% to 94% (Table 2). The survival of ticks injected with dsRNAs or the control was lower in the acquisition (30% to 60.5%, Table 2) compared to the transmission feeding. Such a difference could be due to one of the following: 1) fresh molted adult *R. microplus* ticks are fragile, and the injection procedure is most likely the reason for a poor survival rate; and 2) after acquisition feeding, ticks were forcibly removed from infected calf and transferred to naïve animals for transmission feeding. During this manipulation, the tick mouthpart might be harmed, which could prevent tick re-attachment.

**Figure 3:**
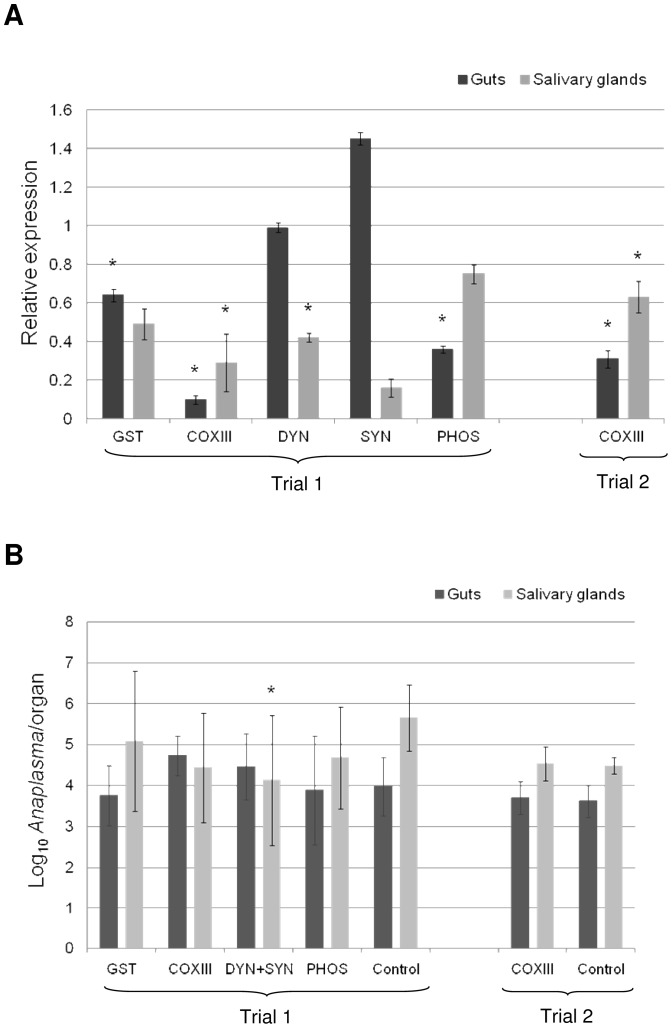
Gene knockdown efficiency and *A. marginale* levels on dsRNA injected ticks after transmission feeding. (A) The expression of the cytochrome *c* oxidase sub III (COXIII), glutathione S-transferase (GST), synaptobrevin (SYN), dynein (DYN) and phosphatidylinositol-3,4,5-triphosphate 3-phosphatase (PHOS) genes in guts and salivary glands from dsRNA injected and control ticks (injection of an equal volume of 0.1 mM EDTA) were evaluated by RT-qPCR. The relative expression level of each gene in dsRNA-injected ticks in relation to EDTA-injected ticks (control) was calculated by the Delta Delta Ct method. Threshold values were normalized according to the Ct of the reference gene (tubulin). The data represent the mean ± S.D. of four pools of 5 guts and salivary glands. An asterisk (*) represent data with differences statistically significant with respect to control (*P*≤0.05). (B) The bacteria number was determined by qPCR using specific primers and a TaqMan probe for *msp*5 gene.

The efficiency of gene knockdown on GST, COXIII, DYN, SYN and PHOS transcript levels was assessed in the guts and salivary glands after tick transmission feeding (Figure 3A). The relative gene expression of GST, COXIII and PHOS was significantly lower than the control in guts, presenting values of 0.64±0.034, 0.1±0.023 and 0.36±0.02, respectively. However, in salivary glands from ticks injected with dsCOXIII, dsDYN and dsSYN, the relative gene expression was 0.29±0.148, 0.42±0.023 and 0.16±0.048, respectively, which were significantly lower than in the control.

The bacteria infection was evaluated in guts and salivary glands from dsRNA-injected and control ticks after transmission feeding. Knockdown of tick genes resulted in no reduction of bacterial loads during transmission feeding, except in salivary glands injected with dsDYN+SYN (Figure 3B).

**Figure 4:**
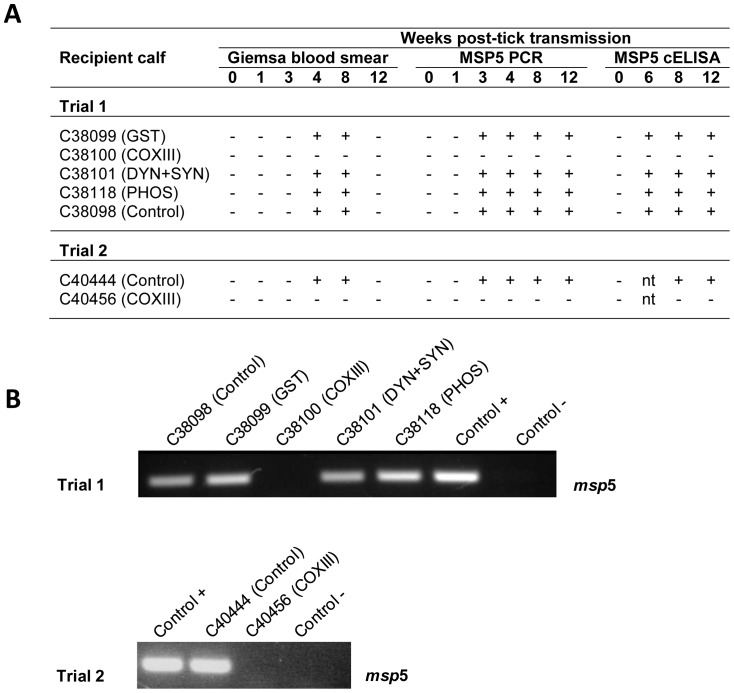
Impact of tick gene knockdown on *A. marginale* transmission to calves. (A) Detection of *A. marginale* in the blood of calves used as hosts for infected tick feeding at multiple time points. Recipient calves (represented by numbers) received specific dsRNA injected ticks. Giemsa blood smear: microscopic examination of a minimum of 50 high-power fields. MSP5n PCR: amplification of *msp*5 by nPCR. MSP5c ELISA: Competitive inhibition enzyme-linked immunosorbent assay for MSP5 protein detection. nt: not tested. (B) Visualization of MSP5 nPCR products from calves blood (panel A) from trials 1 and 2 at the end of the 12^th^ week by agarose gel electrophoresis stained with SYBR Safe (Invitrogen). Control +: blood from an *Anaplasma*-infected calf. Control**-**: without template DNA.

After the removal of ticks, recipient calves were monitored weekly for up to 12 weeks post-tick transmission feeding. All recipient calves were positive for *A. marginale* infection by Giemsa-stained blood smear analysis within the 4^th^ week post-tick feeding, except the calf that received ticks injected with dsCOXIII (Figure 4A). Similar results were obtained by nested PCR (nPCR) during the 3^rd^ week (Figure 4A). The visualization of the MSP5 nPCR product from blood samples at the end of the 12^th^ week by electrophoresis is shown in Figure 4B.

Furthermore, all calves used in transmission studies seroconverted at the 6^th^ week post-tick feeding (Figure 4A), with the exception of the calf that received ticks injected with dsCOXIII. The values of the peak bacteria and the package cell volume (PCV) were similar for all experimental calves: for calf C38098 (control calf), the peak bacteremia was 4.8% and PCV 22% at the 6^th^ week; for calf C38099 (dsGST), the peak bacteremia was 8.0%, PCV 21% at the 7^th^ week; for calf C38101 (dsDYN+SYN), the peak bacteremia was 2.6%, PCV 23% at the 7^th^ week; and for calf C38118 (dsPHOS), the peak bacteremia was 3.6%, PCV 20% at the 7^th^ week.

#### Trial 2

Due to failure of *A. marginale* transmission to calf by ticks with COXIII knockdown in the trial 1, a new batch of experiments was set up with dsCOXIII-injected and control ticks. In the second trial, ticks engaged in acquisition feeding on a calf (C39306) with a bacteremia ranging from 0.05% to 6.8% and a PCV of 30% to 38%. There was no significant difference in tick survival rates between the dsCOXIII-injected ticks (41%) and control ticks (39%) after acquisition feeding (Table 2). Similarly, at the end of transmission feeding, no difference was observed between the tick survival rate in dsCOXIII-injected ticks (88%) and control ticks (79%) (Table 2). The COXIII gene knockdown was efficient because the relative gene expression was significantly lower than the control both in the gut (0.31±0.045) and salivary glands (0.63±0.082) (Figure 3A). However, there was no difference between dsCOXIII-injected and control ticks with respect to the *Anaplasma* level in the gut and salivary gland (Figure 3B).

Similarly to the previous trial, the recipient calf (C40456) that received ticks injected with dsCOXIII remained negative by Giemsa-stained blood smears, nPCR and MSP5-CI-ELISA at all-time points tested (Figure 4A), while *A. marginale* was detected by a Giemsa-stained blood smear from the blood of the calf (C40444) within the 4^th^ week post-feeding of control ticks and confirmed by nPCR at the 3^rd^ week. The detection of MSP5 nPCR product from the blood samples at the end of the 12^th^ week is shown in the Figure 4B. The calf seroconverted at the 8^th^ week post-tick transmission and had a peak bacteremia of 8.0% and PCV 26% at the 7^th^ week (Figure 4A).

## Discussion

In this study, we initially identified a set of *R. microplus* genes that are modulated in response to *A. marginale* infection in two suppression-subtractive hybridization libraries using a BME26 cell line as model. Subsequently, we tested if knocking down selected up-regulated tick genes could affect *A. marginale* infection at the level of the tick gut and salivary glands. In addition, the *A. marginale* infection was evaluated in recipient calves that received gene knocked-down ticks.

We annotated by function 37 tick genes that were up-regulated in response to *A. marginale* infection on BME26 cells (Table S2). Some of these genes have been identified in other “omics” studies [12], [16], [44]. A proteomic and a transcriptomic approach using IDE8 cells identified the over-expression of several genes, including glutathione S-transferase (GST), cytochrome *c* oxidase subunit II and selenoprotein due to *A. marginale* infection, which is similar to our findings [12]. Furthermore, a temporal study of gene expression in adult male *R. microplus* in response to *A. marginale* infection identified several up-regulated genes, including those similar to our library, such as GST and aldehyde dehydrogenase [44]. Finally, a transcriptomic study comparing uninfected and *A. marginale*-infected *R. microplus* salivary glands found induction of the cytochrome *c* oxidase subunit III (COXIII) and a proline-rich protein [16], as identified in our library. However, other genes identified in this previous study, such as the vacuolar H+-ATPase V1 sector subunit, metallothionein, glycine-rich proteins and the von Willebrand factor, have not been detected in the current study. Differences between our results and Zivkovic et al [16] results could be due to 1) the use of an embryonic cell line as opposed to infected tick organs, 2) divergence of *A. marginale* strains, 3) differences in experimental infection conditions used between the studies

After an accurate analysis of up-regulated BME26 genes, five genes were selected for a functional genomic study based on their biological role and putative connection with *Anaplasma* colonization: GST is involved with detoxification processes and promotes cell protection against oxidative stress; COXIII is associated with mitochondrial metabolism that results in ATP or reactive oxygen specie (ROS) generation; DYN and SYN are related to intracellular trafficking pathways and may be associated with bacteria survival and replication within tick cell vacuoles; and PHOS is involved in several cellular functions, such as cytoskeleton reorganization and NADPH and ROS production. Among these genes, only GST and COXIII have been previously shown to be up-regulated in cell culture and in ticks in response to *A. marginale* infection and during blood feeding [12], [15], [16], [44]–[46]. The over-expression of GST in IDE8 tick cells and *D. variabilis* salivary glands by *A. marginale* infection was suggested as a response to reduce the oxidative stress caused by pathogen infection and thus increases pathogen multiplication in tick cells [12]. In ISE6 tick cells, GST was also up-regulated [15]. After nine days of *A. marginale* infection, a higher amount of GST was expressed in the *R. microplus* gut than in the salivary gland [44]. A similar modulation of the expression profile was observed for the COXIII gene in IDE8 cells in response to *A. marginale* infection [12], and the up regulation was also verified in the salivary glands of adult male *R. microplus* [16], [44]. Up-regulation of cytochrome *c* oxidase during pathogen infection could be associated with an increase in the metabolic demands placed on arthropod vectors by the pathogens. A previous study demonstrated that cytochrome *c* oxidase subunit VIb was up-regulated in *Anopheles gambiae* upon *Plasmodium berghei* infection [45]. COX subunit I gene expression was also induced after a blood meal in the *Ixodes ricinus* gut, salivary gland and hemolymph [46].

The silencing of the five selected genes resulted in no reduction in bacterial loads during transmission feeding, except in salivary glands from dsDYN+SYN-injected ticks. In contrast with our results, a previous study demonstrated that the knockdown of dsGST in *D. variabilis* resulted in a reduction of the *A. marginale* infection in the gut and salivary glands [47]. These differences in outcome could be associated with the use of different *A. marginale* strains and/or different species of ixodid ticks.

In addition to the impact of the gene knockdown on tick infection, we were interested in evaluating the transmission of *A. marginale* to calves after tick gene silencing. Interestingly, only the ticks injected with dsCOXIII failed to transmit *A. marginale* as determined by serology, PCR and Giemsa stained blood smears in two distinct trials. The monitoring of *Anaplasma* in the blood of the calves was performed for up to 12 weeks after tick feeding without finding any sign of infection. Previous studies using similar experimental conditions demonstrated that 25–30 days post-tick feeding is the period that elapses between the ingress of the etiologic agent and the emergence of the earliest detectable forms of such agents [48], [49]. The absence of *Anaplasma* infection in the calves that received dsCOXIII ticks could not be due to animal age because animals of any age can be infected, and the severity of disease is age-dependent [48]. We propose two biological explanations for the lack of *A. marginale* transmission by ticks injected with dsCOXIII: (i) COXIII knockdown interfered with the release of *A. marginale* into saliva either completely or below the transmission threshold and/or (ii) the gene knockdown affected *A. marginale* viability. Experiments are being conducted to elucidate how COXIII knockdown affects *A. marginale* transmission to calves.

Cytochrome *c* oxidase (COX) or complex IV is the terminal enzyme of the mitochondrial electron transport chain. The subunit III of COX (COXIII) is encoded by the mitochondrial genome and, with COXI and COXII, constitutes the COX catalytic core [50]. COX drives electrons that flows from cytochrome *c* to molecular oxygen and promotes the proton pump to the intermembrane space that is used to produce ATP. In addition, mitochondrial oxidative metabolism is also a major source of cellular ROS. One consequence of a poorly functioning COX is a reduction in ATP generation. The release of *Anaplasma* into tick saliva may be compromised because it has been postulated that *A. marginale* exit from tick cells by fusing the colony with the cell membrane [51], which requires ATP. Another effect of COX malfunction is the large production of ROS, resulting in cellular damage [52]. In yeast cells, partially assembled cytochrome *c* oxidase produces increased ROS levels that block proliferation of the cells [53]. Such increased levels of ROS due to a dysfunctional cytochrome *c* oxidase could affect *A. marginale* viability and consequently its transmission to the calves.

Previous studies have demonstrated that the knockdown of several tick genes, including subolesin [13], [47], [54], [55], GST [47], salivary selenoprotein M [47], H+ transporting lysosomal vacuolar proton pump [47] and varisin [56], reduce *A. marginale* levels in the gut and salivary gland. However, none of these studies evaluated the effect of gene silencing on bacterial transmission to naïve calves. The data presented here showed clearly that COXIII knockdown provoked a failure of *A. marginale* transmission to calves even while it did not affect the bacterial load in the ticks. This is the first report demonstrating that knockdown of a tick gene is associated with a miscarriage of *A. marginale* transmission.

## Supporting Information

Table S1 **Primer sets for RT-qPCR and dsRNA.**
(DOC)

Table S2 **Hyperlinked excel spreadsheet with assembled up and down contigs from SSH libraries and details of the sequence match.**
(XLSX)
