# Correction: Knockdown of the *Rhipicephalus microplus* Cytochrome *c* Oxidase Subunit III Gene Is Associated with a Failure of *Anaplasma marginale* Transmission

**DOI:** 10.1371/journal.pone.0106877

**Published:** 2014-09-08

**Authors:** The PLOS ONE Staff

There is an error in the title of the y axis of Figure 2. Please see the corrected Figure 2 here.

**Figure 2:**
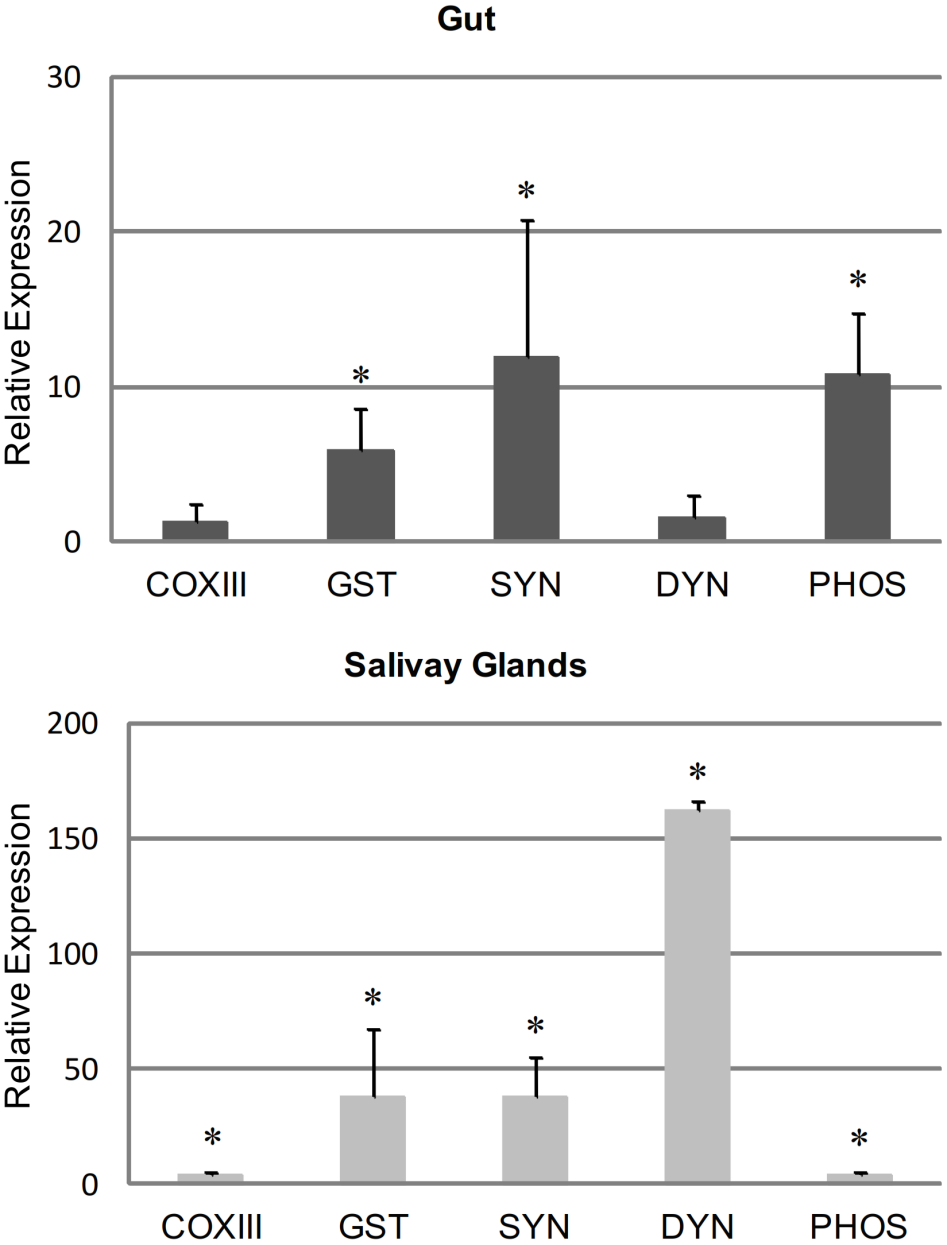
Relative gene expression in gut and salivary glands of ticks infected with *A. marginale*. The expression of the cytochrome *c* oxidase sub III (COXIII), glutathione S-transferase (GST), synaptobrevin (SYN), dynein (DYN) and phosphatidylinositol-3,4,5-triphosphate 3-phosphatase (PHOS) genes in guts and salivary glands from *R. microplus* males fed for 8 days on either one uninfected calf (C38080) or one *A. marginale*-infected calf (C37837) was assessed by RT-qPCR. Threshold values were normalized according to the Ct of the reference gene (tubulin). The relative expression level of each gene in infected ticks in relation to uninfected ticks (control) was calculated using the Delta Delta Ct method. The data represent the mean ± S.D. of four pools of 5 guts and salivary glands. An asterisk (*) represent data with differences statistically significant with respect to control (*P*≤0.05).
