# Supplementary material for: Knockdown of the Rhipicephalus microplus Cytochrome c Oxidase Subunit III Gene Is Associated with a Failure of Anaplasma marginale Transmission
Source: PLoS One. 2014 May 30;9(5):e98614. doi: 10.1371/journal.pone.0098614 (PMC4039488; doi:10.1371/journal.pone.0098614)
Supplement: Table S1 — Primer sets for RT-qPCR and dsRNA. (DOC) [file pone.0098614.s001.doc]

**Table S1. Primer sets for RT-qPCR and dsRNA**

| **Gene** | **RT-PCR primer sequences** | **dsRNA primer sequences** |
| --- | --- | --- |
| GST | AGCAGCCAATGGCAACATTA  TTCGCACTCCTTCACCTCAT | CGGTCCTGCTGTACAACCTT  AATGGCATTGCTCTCGTACA |
| COXIII | TTCATGTGTTAATTGGTTCAATTTTT  GCAGAAGCTTCAAAGCCAAA | GGCTTTGAGTATTTTCAAGCTCA  AAAAATTGAACCAATTAACACATGAA |
| DYN | ACATAGCGGCCTACATCAAGA  TCTCGTGGGTGACGTAGGAT | CAGGAAGGCAGTCATCAAGA  CCAGGTGGGGTTGTACTTCT |
| SYN | CTCGATCCACCATTGAAACA  GCCTCTTCGAAAACGACTTCT | TGGCAACAAGGTCAATTCTG  GTTGAGCGCAAATGCAGATA |
| PHOS | AGGCACTGGAGAGTTCCTGA  TTTCGACGGACACATAGGC | TTGACCAAAGGAGCCAGACT  TTTCGGGATCCTCATTCAAG |
| Tubulin | CGTGCCGTATTTGTTGATC  AGATTAGCTGCTCCGGGTG |  |
